# Addressing presbyopia in rural central India: the Sadguru experience

**Published:** 2026-03-12

**Authors:** Subeesh Kuyyadiyil, Elesh Jain, Ujjal Bhattacharya, Pushependra Tripathi

**Affiliations:** 1 Head, Community Opthalmology: Shri Sadguru Seva Sangh Trust, Chitrakoot, India.; 2 CEO & Trustee: Shri Sadguru Seva Sangh Trust, Chitrakoot, India.; 3 Consultant - Grants and Programme Director, Vision Care Initiative: Shrimad Rajchandra Love and Care, Chitrakoot, India.; 4 Project Associate: Shri Sadguru Seva Sangh Trust, Chitrakoot, India.

**Near-vision screening and spectacle dispensing were successfully integrated into existing outreach and vision centre services.**

**Figure:**
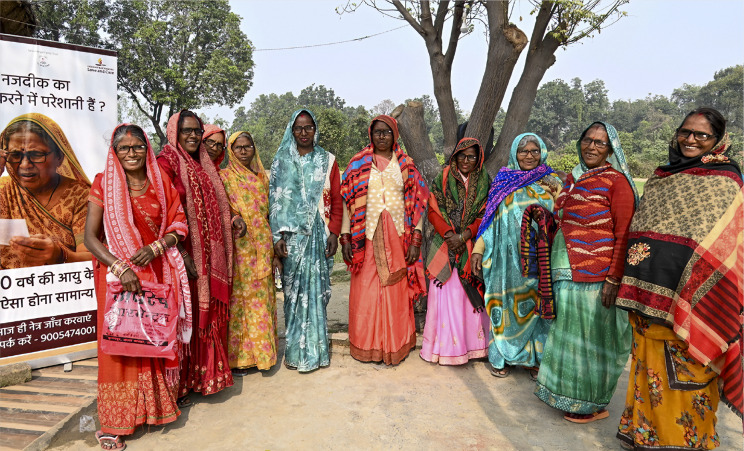
Women wearing near-vision spectacles provided after presbyopia screening at a community-based eye care camp. INDIA

© SADGURU CC BY-NC-SA 4.0

Presbyopia is an age-related loss of accommodation that reduces near vision and interferes with everyday tasks.^1,2^ National estimates indicate that about one-third of Indian adults aged ≥30 years have uncorrected presbyopia, corresponding to roughly 170-180 million people.^3^ In Madhya Pradesh and Uttar Pradesh, where eye care remains limited and largely urban-centred,^4^ this burden falls disproportionately on rural adults in their forties and fifties who rely on good near vision for livelihood and household activities such as reading farm records, stitching, sorting grains, counting money, and using mobile phones. Within this context, Sadguru Netra Chikitsalaya's rural eye-care programme records have consistently identified presbyopia as a leading cause of near-vision impairment at the primary-care level, exceeding early cataract, uncorrected hyperopia, and other causes.

## Rationale, planning, and strategy

Presbyopia is common in rural India and spectacle coverage remains low, resulting in a substantial unmet need.^6^ Ready-made near vision spectacles are inexpensive and can restore functional vision immediately; presbyopia therefore represents a high-burden condition with an uncomplicated, scaleable solution. With support from Sarvamangal Family Trust, through the Shrimad Rajchandra Love and Care Programme, the Sadguru team have repositioned presbyopia from a secondary concern to a strategic programme priority by setting up the Vision Care Project joint initiative to conduct community-based presbyopia screening and provide free, ready-made near-vision spectacles in under-served rural areas. The initiative was specifically designed to integrate near-vision services into existing, routine services, without the need for additional infrastructure or staffing.

“Ready-made near vision spectacles are inexpensive and can restore functional vision immediately; presbyopia therefore represents a high-burden condition with an uncomplicated, scaleable solution.”

Programme planning was informed by outreach experience and service data. Teams observed that roughly one-third of adults attending cataract camps in Satna, Rewa, and Chitrakoot primarily reported near-vision difficulty rather than cataract-related symptoms. Camp and vision centre records confirmed this pattern across more than 20 outreach blocks, with historically low access to refractive services.

Rather than creating a separate, vertical programme, Sadguru launched a pilot programme that involved strengthening existing outreach and vision centre platforms in four villages in Satna district.

The pilot included three models:

- **Outreach-based presbyopia camps.** Simple awareness messages about near-vision screening at existing eye camps were promoted via Sadguru's established rural outreach network, consisting of village leaders, self-help groups, and local NGOs. Rapid testing and same-day dispensing were found to be particularly effective for working adults and for women who may not be independent in seeking eye care, due to social barriers, limited literacy, or lack of confidence to travel alone.
- **A vision centre ‘near-vision first’ pathway.** To increase walk-ins at vision centres, a presbyopia-focused communication package was introduced. Banners, posters, and leaflets in markets and panchayat offices encouraged adults to attend for a quick near-vision check. This simple message increased self-referrals and helped normalise presbyopia care within routine vision centre services.
- **Screening at large religious and community gathering.** Temples, religious fairs, and festivals were used to reach older adults who were reluctant to visit clinics. This approach was efficient for high-volume mobilisation and first-time spectacle provision; it was less suited to systematic follow-up.

All three models in the pilot programme followed the same standardised clinical protocol as part of a structured screening and triage pathway, which ensured that near-vision spectacle dispensing remained embedded within comprehensive eye care.

- Adults aged 35 years and older underwent near-vision testing using an N-chart at 40 cm, a minimum distance-vision check (using a Snellen or E chart), and a torchlight anterior-segment examination.
- Adults whose vision improved with plus lenses (+1.00 D to +3.50 D) and who showed no signs of ocular pathology were classified as having uncomplicated presbyopia and received ready-made readers there and then.
- Those with reduced distance vision, persistent symptoms, or suspected ocular disease were counselled and referred (through established outreach mechanisms) to vision centres, where they underwent full refraction, slit-lamp examination, and intraocular pressure assessment, with onward referral to the base hospital when indicated.

Early findings of the pilot programme shaped the final strategy. Same-day dispensing, convenient scheduling aligned with self-help group meetings and weekly markets, and simple local messaging around “*nazdeek ka kaam*” (“near work”) markedly increased uptake. Mobilisation through community leaders and trained health volunteers improved women's participation and enabled rapid, reliable delivery of presbyopia care within routine rural eye health services.

## Procurement, pricing, and quality assurance

Spectacles were centrally procured to ensure a reliable supply chain. A standard range of plus lenses (+1.00 D to +3.50 D) simplified logistics while covering most people's needs. Each batch underwent checks for lens power, frame strength, and scratch resistance, and defective lots were returned to suppliers. Depending on the setting and funding support, spectacles were provided free of cost or at a nominal subsidised price (approximately INR 100-200 per pair) to maintain affordability and minimise financial barriers.

## Results and measurable outcomes

Over ten months, the programme screened 824,769 adults across 24 districts in Uttar Pradesh and Madhya Pradesh. Of those screened, 37% reported near-vision difficulty. Among those screened, about 21.4% had uncomplicated presbyopia requiring ready-made readers, while the remainder had early cataract, uncorrected refractive error, or other ocular disease and were referred appropriately. After adjustments in timing and messaging, women comprised around 45% of spectacle recipients. In total, 176,543 read-made spectacles were dispensed on the same day.

A brief compliance survey of approximately 5% of recipients (around 8,800 of those who received spectacles), was conducted through phone interviews and selected field visits. This indicated that more than 76% of recipients continued to use their spectacles at three months. Users reported improved ability to read, stitch, maintain accounts, and use mobile phones, with perceived gains in both productivity and quality of life.

## Patient vignette

Vidya Namdev (name changed), 56-year-old tailor, had begun losing customers as her stitches became uneven, which she assumed was an unavoidable consequence of ageing. At a presbyopia camp, she was found to have reduced near vision and was provided with +1.50 D readers. She experienced immediate improvement and, with restored near vision, her stitching accuracy, speed, and confidence returned, allowing her to retain clients and income.

## Key lessons and recommendations

Integrating presbyopia services into existing outreach and vision centre platforms was efficient, scalable, and cost-effective, and did not require major new infrastructure. Using the existing outreach network meant that a separate stand-alone system was unnecessary. Simple awareness messages, convenient scheduling, particularly for women, on-the-spot dispensing, and centralised, quality-assured procurement were critical for high uptake and sustained use.

Programmes in similar rural settings can replicate this model by incorporating routine near-vision screening into existing eye care activities, maintaining a basic stock of ready-made readers at outreach camps and vision centres, and mobilising communities through local leaders and frontline workers. Providing spectacles free of cost, or at low cost, supports rapid adoption and helps reduce the large burden of uncorrected presbyopia in low-resource settings.
